# Supplementary material for: Causal Relationship between Aging and Anorexia Nervosa: A White-Matter-Microstructure-Mediated Mendelian Randomization Analysis
Source: Biomedicines. 2024 Aug 16;12(8):1874. doi: 10.3390/biomedicines12081874 (PMC11351342; doi:10.3390/biomedicines12081874)
Supplement: Supplementary file 1 [file biomedicines-12-01874-s001.zip › Supplementary Figure/Figure S4/Singe SNP plot of MR analysis for TL to ubm-b-1936.pdf]

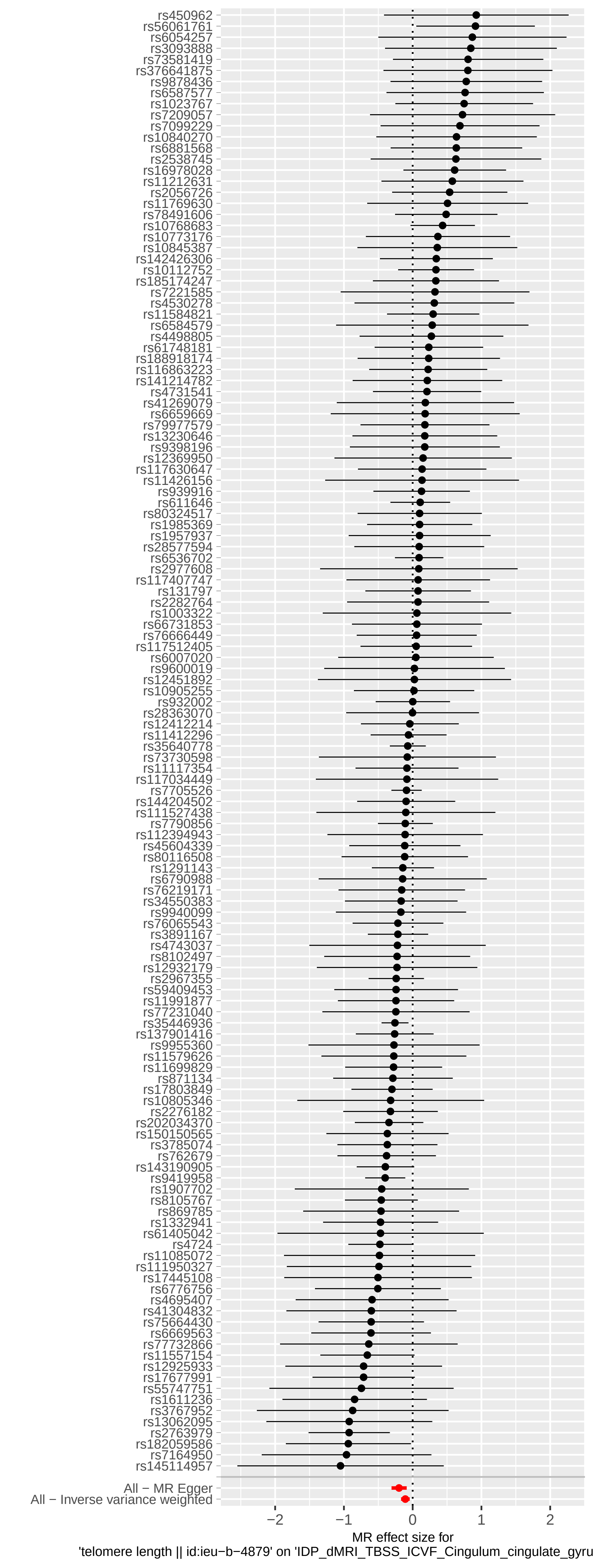

rs450962
rs56061761
rs6054257
rs3093888
rs73581419
rs376641875
rs9878436
rs6587577
rs1023767
rs7209057
rs7099229
rs10840270
rs6881568
rs2538745
rs16978028
rs11212631
rs2056726
rs11769630
rs78491606
rs10768683
rs10773176
rs10845387
rs142426306
rs10112752
rs185174247
rs7221585
rs4530278
rs11584821
rs6584579
rs4498805
rs61748181
rs188918174
rs116863223
rs141214782
rs4731541
rs41269079
rs6659669
rs79977579
rs13230646
rs9398196
rs12369950
rs117630647
rs11426156
rs939916
rs611646
rs80324517
rs1985369
rs1957937
rs28577594
rs6536702
rs2977608
rs117407747
rs131797
rs2282764
rs1003322
rs66731853
rs76666449
rs117512405
rs6007020
rs9600019
rs12451892
rs10905255
rs932002
rs28363070
rs12412214
rs11412296
rs35640778
rs73730598
rs11117354
rs117034449
rs7705526
rs144204502
rs111527438
rs7790856
rs112394943
rs45604339
rs80116508
rs1291143
rs6790988
rs76219171
rs34550383
rs9940099
rs76065543
rs3891167
rs4743037
rs8102497
rs12932179
rs2967355
rs59409453
rs11991877
rs77231040
rs35446936
rs137901416
rs9955360
rs11579626
rs11699829
rs871134
rs17803849
rs10805346
rs2276182
rs202034370
rs150150565
rs3785074
rs762679
rs143190905
rs9419958
rs1907702
rs8105767
rs869785
rs1332941
rs61405042
rs4724
rs11085072
rs111950327
rs17445108
rs6776756
rs4695407
rs41304832
rs75664430
rs6669563
rs77732866
rs11557154
rs12925933
rs17677991
rs55747751
rs1611236
rs3767952
rs13062095
rs2763979
rs182059586
rs7164950
rs145114957
All − MR Egger
All − Inverse variance weighted
−2
−1
0
1
2
MR effect size for
'telomere length || id:ieu−b−4879' on 'IDP_dMRI_TBSS_ICVF_Cingulum_cingulate_gyru
